# Bridging the Awareness–Action Gap in Disaster Preparedness: A Qualitative Study of Immersive Virtual Reality Learning Among Nursing Students

**DOI:** 10.1111/nicc.70681

**Published:** 2026-09-24

**Authors:** Keiko Mori, Miina Yamazaki, Yoshiyuki Haruna

**Affiliations:** ^1^ Graduate School of Health Sciences Okayama University Okayama Japan; ^2^ Kobe University Hospital Kobe Japan; ^3^ Snowlion. Inc. Okayama Japan

**Keywords:** awareness–action gap, critical care nursing, disaster preparedness, disaster risk perception, nursing students, simulation‐based education, virtual reality

## Abstract

**Background:**

Disasters increasingly challenge healthcare systems, highlighting the need to strengthen preparedness among future healthcare professionals. Although disaster risk awareness is important, it does not consistently translate into preparedness behaviours, reflecting the awareness–action gap. Immersive virtual reality (VR) may enhance experiential disaster learning, but how it influences preparedness processes remains insufficiently understood, particularly in healthcare and critical care education.

**Aims:**

To explore how immersive VR‐based disaster simulations influence disaster risk perception and preparedness among nursing students, with particular attention to the awareness–action gap and implications for future healthcare and critical care practice.

**Study Design:**

A qualitative descriptive study using immersive VR‐based disaster simulations followed by semi‐structured interviews. Twelve undergraduate and graduate nursing students in Japan experienced immersive VR simulations depicting floods and landslides. Individual semi‐structured interviews explored emotional responses, disaster risk perception, preparedness awareness and anticipated behavioural responses. Data were analysed using Krippendorff's content analysis with researcher triangulation.

**Results:**

Five categories were identified: (1) heightened vulnerability and psychological burden through immersion; (2) persistence of the awareness–action gap despite increased risk perception; (3) enhanced risk recognition and preparedness intentions; (4) recognition of the need for social and structural support systems; and (5) emerging responsibility accompanied by perceived limitations. VR transformed abstract disaster risk into emotionally salient experiences, strengthening threat appraisal and preparedness awareness; however, awareness did not consistently translate into preparedness behaviours, reflecting insufficient coping appraisal and self‐efficacy.

**Conclusions:**

Immersive VR may strengthen disaster risk perception and preparedness awareness, but immersive experiences alone may be insufficient to bridge the awareness–action gap. Combining VR with reflective learning, skills‐based training and collaborative simulation may strengthen coping appraisal and preparedness action.

**Relevance to Clinical Practice:**

Immersive VR combined with reflective and skills‐based learning may support psychological readiness, situational awareness and disaster preparedness competencies among future nurses, including those preparing for critical care practice.

## Impact Statements

- What is known about the topic
  ○ Disaster preparedness among healthcare professionals is essential for effective responses to disasters and critical care emergencies.
  ○ Immersive virtual reality can provide realistic and emotionally engaging disaster learning experiences.
  ○ Increased disaster risk awareness does not necessarily translate into preparedness behaviours, reflecting an awareness–action gap.
- What this paper adds
  ○ Immersive virtual reality transformed abstract disaster risks into personally salient experiences, strengthening nursing students' risk perception and preparedness awareness.
  ○ Increased risk perception alone did not consistently lead to preparedness action, suggesting that coping appraisal and self‐efficacy are important mechanisms underlying the awareness–action gap.
  ○ Integrating immersive virtual reality with reflective learning, skills‐based training and collaborative simulation may better support preparedness competencies relevant to disaster and critical care nursing.

## 1 Introduction

Disasters are increasing in frequency, intensity and complexity worldwide, largely due to climate change, urbanisation and population vulnerability (United Nations Office for Disaster Risk Reduction [1]; World Health Organization [2]). These events place substantial pressure on healthcare systems and often result in mass casualties, disruption of infrastructure and shortages of healthcare resources. Critically ill and medically vulnerable populations are particularly affected during disasters because of interruptions in emergency response, evacuation challenges and reduced access to intensive care services. Consequently, strengthening disaster preparedness has become an important international priority for healthcare systems and healthcare professionals.

Healthcare professionals, particularly nurses, play essential roles in disaster response, including emergency care provision, coordination of resources and psychosocial support [3, 4]. In disaster situations, critical care nurses are frequently required to respond to rapidly deteriorating patients under conditions of uncertainty, limited resources and high psychological burden [5]. Critical care settings may also experience increased patient acuity and surge capacity challenges during mass casualty incidents and large‐scale disasters. Therefore, preparing nursing students for disaster situations is closely related to strengthening future critical care preparedness and healthcare system resilience.

Previous studies have demonstrated that although nursing students are generally aware of disaster risks, this awareness does not necessarily translate into preparedness behaviours [6, 7, 8]. This phenomenon reflects the widely recognised awareness–action gap in disaster preparedness [9]. In addition, students without prior disaster experience tend to perceive disasters abstractly, limiting their ability to anticipate real‐life risks and respond effectively [10, 11].

Traditional disaster education methods, such as lectures and evacuation drills, are effective for knowledge acquisition but often lack realism and emotional engagement, which are essential for promoting behavioural change [12]. Furthermore, conventional educational approaches may not adequately prepare nursing students for the psychological stress, rapid decision‐making and uncertainty associated with disaster and critical care situations. In recent years, immersive technologies such as virtual reality (VR) have emerged as innovative educational tools that enable experiential learning by simulating realistic disaster environments.

Emerging evidence suggests that VR‐based disaster education can enhance risk perception and preparedness intentions [13, 14, 15]. Immersive simulation‐based learning has also gained increasing attention within emergency and critical care nursing education because it may support situational awareness, emotional preparedness and decision‐making in high‐pressure clinical environments. From a theoretical perspective, these effects can be understood in relation to Protection Motivation Theory, which highlights the roles of threat appraisal and coping appraisal in motivating protective behaviours [16].

However, despite these promising findings, there remains limited qualitative research exploring how immersive VR experiences influence disaster risk perception and preparedness processes, particularly the mechanisms underlying the awareness–action gap. In addition, little is known about how immersive disaster education may contribute to preparedness among future nurses who may later respond to disaster‐related critical care situations.

## 2 Aims

Therefore, this study aimed to explore how immersive VR‐based disaster simulations influence disaster risk perception and preparedness among nursing students, with a particular focus on the awareness–action gap. This study also sought to examine the potential implications of immersive VR‐based learning for disaster preparedness education and future critical care nursing practice.

## 3 Design and Methods

### 3.1 Study Design

A qualitative descriptive design was employed to explore how immersive VR‐based disaster experiences influence disaster risk perception and preparedness among nursing students, with a particular focus on the awareness–action gap. A qualitative descriptive approach was considered appropriate because this study aimed to obtain rich descriptions of participants' experiences and perceptions following immersive disaster simulations [17]. The study is reported in accordance with the Consolidated Criteria for Reporting Qualitative Research (COREQ).

### 3.2 Participants and Setting

Participants were nursing students enrolled in undergraduate or graduate nursing programmes at a university in Japan. The study was conducted at a university that provides both undergraduate and graduate nursing education. Disaster nursing is included within the nursing curriculum, providing students with opportunities to learn about disaster preparedness and nursing roles in disaster settings. The study was conducted in a university‐based educational setting rather than in a clinical or disaster response setting. Eligibility criteria included: (1) enrolment in a nursing programme, (2) ability to participate in a VR‐based disaster simulation, and (3) provision of informed consent. No specific exclusion criteria were established beyond these eligibility criteria. All participants who met the eligibility criteria and provided informed consent completed both the VR‐based disaster simulation and the subsequent interview; therefore, no participants were excluded from the study. A recruitment notice was posted on the university's online learning management system. Students who were interested in disaster nursing and wished to participate voluntarily contacted the researchers directly. The researchers then provided them with detailed information about the study, and written informed consent was obtained from those who agreed to participate. Purposive sampling was used to recruit participants with varying academic levels and educational experiences in order to obtain diverse perspectives regarding disaster preparedness and immersive learning experiences. Data collection was conducted in a private room within the university.

### 3.3 Data Collection

Data were collected between October and November 2025. Participants experienced immersive VR disaster content developed by Snowlion Inc. (Okayama, Japan) using a VR headset. The VR content depicted flood and landslide disaster scenarios and was designed to recreate emotionally engaging and realistic disaster environments in which participants experienced evacuation‐related uncertainty, environmental danger, and time pressure. The entire VR session, including both the flood and landslide scenarios, lasted approximately 30 minutes.

Interviews lasted approximately 30–60 min, were audio‐recorded with consent and transcribed verbatim. Field notes were recorded following each interview to capture contextual observations and researchers' reflections during data collection.

### 3.4 Data Analysis

Data were analysed using Krippendorff's approach to content analysis [18]. Meaning units were identified, coded and grouped into subcategories, which were subsequently synthesised into categories through cross‐case comparison. Multiple researchers independently analysed the data and engaged in iterative discussions to reach consensus. This analytical approach enabled systematic exploration of participants' perceptions, emotional responses and preparedness‐related experiences associated with immersive VR learning.

### 3.5 Rigour

Trustworthiness was ensured through credibility, dependability and confirmability [19]. Credibility was enhanced through researcher triangulation, dependability through maintaining an audit trail and confirmability by grounding interpretations in participants' narratives. To enhance analytical rigour, coding and category development were repeatedly reviewed through collaborative discussions among the research team. Data collection continued until thematic saturation was achieved.

### 3.6 Ethical Considerations

Before the commencement of data collection, the study underwent a simplified ethical review process at Okayama University, and approval to conduct the study was obtained. Under institutional procedures, this approval permitted the conduct of the study but did not permit external dissemination of the findings. Therefore, following completion of data collection, the study and its findings underwent additional formal review, and ethical approval for external dissemination was obtained post‐hoc from the Ethics Committee of Okayama University Graduate School of Health Sciences (Approval No. OUHS2025‐0087F, 26 March 2026). Written informed consent was obtained from all participants before participation. All data were anonymised and securely stored.

## 4 Findings

### 4.1 Participant Characteristics

Participant characteristics are presented in Table 1. A total of 12 nursing students participated in this study, including first‐, third‐ and fourth‐year undergraduate students as well as graduate students. Interview durations ranged from 29 to 46 min. All interviews were conducted face‐to‐face in a private setting and were audio‐recorded with participants' consent.

**TABLE 1:** Participant characteristics.

Participants	Academic year	Age (years)	Gender	Interview duration (min)	Prior disaster experience
A	Third‐year undergraduate student	20	Female	32	No
B	Third‐year undergraduate student	20	Female	31	No
C	First‐year undergraduate student	18	Female	40	No
D	First‐year undergraduate student	18	Female	30	No
E	Fourth‐year undergraduate student	21	Female	38	No
F	Fourth‐year undergraduate student	21	Female	36	No
G	Fourth‐year undergraduate student	21	Female	30	No
H	Fourth‐year undergraduate student	22	Female	38	No
I	Fourth‐year undergraduate student	22	Female	30	No
J	Master's student (Year 2)	26	Female	40	No
K	Master's student (Year 2)	28	Male	35	No
L	Master's student (Year 1)	39	Female	46	No

### 4.2 Overview of Findings

The analysis yielded 118 codes, which were grouped into 10 subcategories and further synthesised into five overarching categories describing how VR‐based disaster experiences influenced disaster risk perception and preparedness (Table 2). The findings illustrate a dynamic process in which immersive VR experiences evoked emotional and cognitive responses that influenced participants' perceptions of vulnerability, preparedness awareness and perceived disaster response capabilities. Participants also reflected on their future professional responsibilities in disaster and emergency care situations.

**TABLE 2:** Categories and subcategories describing changes in disaster risk perception and preparedness following immersive VR experience.

Category	Subcategory
Heightened vulnerability and psychological burden through immersion	Awareness of vulnerability and psychological burden in extreme situations
	Emotional reactions triggered by immersive and visual stimuli
Fluctuating risk perception and the awareness–action gap	Increased awareness without corresponding behavioural change (awareness–action gap)
	Habituation and reduced sense of urgency through repeated exposure
Enhanced risk recognition and preparedness intentions	Active risk recognition and learning of adaptive behaviours through immersive experience
	Deepened risk perception through emotional and experiential engagement
Recognition of vulnerability and need for support systems	Tension arising from uncertainty about roles and expectations in disaster situations
	Awareness of the need for support systems through imagining vulnerable populations
Emerging sense of responsibility with perceived limitations	Coexistence of motivation to participate and uncertainty about personal capability
	Reinforcement of professional role awareness through experiential learning

### 4.3 Category 1: Heightened Vulnerability and Psychological Burden Through Immersion

This category reflects how immersive VR experiences elicited strong emotional reactions and heightened awareness of personal vulnerability in disaster situations. Participants described feelings of fear, helplessness and difficulty making rapid decisions under extreme conditions. These emotional responses often emerged as participants attempted to navigate uncertain and threatening situations within the immersive environment.

I didn't know which route would be safe… I felt like no matter what I did, it might already be too late (Participant A).

When I imagined it happening in front of me, I felt like my body would freeze and I wouldn't be able to move (Participant D).

Participants further described how the immersive visual and sensory experiences intensified their emotional reactions and created a realistic sense of danger.

The feeling that the tsunami was coming closer and closer was really frightening (Participant E).

It felt suffocating, like I couldn't escape (Participant F).

Several participants also reflected on the psychological pressure associated with making decisions under disaster conditions, which they perceived as relevant to future healthcare and emergency response situations.

### 4.4 Category 2: Fluctuating Risk Perception and the Awareness–Action Gap

This category describes how participants' risk perception increased following the VR experience; however, translating this heightened awareness into concrete preparedness behaviours remained challenging, reflecting the awareness–action gap. Although participants recognised the importance of preparedness, many expressed uncertainty regarding how to initiate or sustain preparedness actions in daily life.

I thought I should prepare an emergency kit, but I still haven't done it (Participant I).

We've never really talked about disasters as a family… maybe we don't feel enough urgency (Participant H).

Participants also described how repeated exposure to disaster‐related information sometimes reduced emotional responsiveness and weakened preparedness motivation over time.

When you keep hearing about disasters, you start to feel less urgency over time (Participant J).

Information can raise awareness, but at the same time, you get used to it and the sense of danger fades (Participant K).

These findings suggest that increased awareness alone may not be sufficient to support sustained preparedness behaviours.

### 4.5 Category 3: Enhanced Risk Recognition and Preparedness Intentions

This category reflects how immersive and emotional experiences facilitated deeper risk recognition and promoted preparedness‐related thinking and behavioural intentions. Participants described how the VR simulations transformed disaster risks from abstract concepts into personally meaningful experiences, encouraging reflection on appropriate disaster responses.

If I watched it a few times, I think I could start thinking more calmly about what to do (Participant L).

You begin to notice what is dangerous and think about how to escape (Participant L).

The immersive nature of the VR experience also appeared to strengthen situational awareness and preparedness‐related thinking.

With VR, it felt like something happening to me, not just something I see on TV (Participant A).

I realised that if I don't act quickly, I might not be able to escape (Participant B).

Several participants further indicated that repeated immersive learning experiences might improve their confidence and preparedness for future disaster situations.

### 4.6 Category 4: Recognition of Vulnerability and Need for Support Systems

This category reflects how awareness of personal and others' vulnerability led participants to recognise the importance of social and structural support systems in disaster situations. Participants became increasingly aware that disaster preparedness extends beyond individual action and requires coordinated community and healthcare support.

I was told that disaster volunteering isn't something easy (Participant D).

It's not something you can just casually do (Participant E).

Participants also reflected on the challenges faced by older adults and vulnerable populations during disasters.

Older people might not evacuate easily, so someone has to help them (Participant H).

I realised how difficult evacuation could be for some people (Participant F).

These reflections encouraged participants to consider the social responsibilities of healthcare professionals during disasters and emergency situations.

### 4.7 Category 5: Emerging Sense of Responsibility With Perceived Limitations

This category describes how VR experiences strengthened participants' sense of responsibility while highlighting uncertainty regarding their own capabilities. Participants expressed growing awareness of their potential future roles as healthcare professionals during disasters, while simultaneously recognising limitations in their current knowledge, confidence and practical abilities.

I want to help, but I don't know what I can actually do right now (Participant I).

I want to be involved, but I'm not sure if I have the ability yet (Participant J).

Participants also described how the VR experience prompted reflection on future nursing and disaster response roles.

If I want to become a nurse, I need to understand disaster preparedness properly (Participant K).

It made me think about how I could help people in a disaster as a healthcare professional (Participant A).

These findings suggest that immersive VR experiences may encourage early professional identity formation related to disaster preparedness and future critical care and emergency response responsibilities.

## 5 Discussion

This study provides insights into how immersive VR‐based disaster experiences influence disaster risk perception and preparedness among nursing students within the broader context of disaster risk reduction (DRR). The findings demonstrated that immersive VR experiences evoked strong emotional engagement, enhanced perceived vulnerability and promoted preparedness awareness and behavioural intentions. However, consistent with previous disaster preparedness research, a persistent awareness–action gap remained, indicating that heightened risk perception alone may be insufficient to produce sustained preparedness behaviours [9, 20]. Importantly, the present study extends existing literature by illustrating how immersive VR‐based learning may contribute not only to disaster preparedness education but also to future healthcare and critical care preparedness. Participants described emotional pressure, uncertainty and rapid decision‐making challenges during the simulations, experiences that may resemble aspects of disaster‐related emergency and critical care situations. These findings suggest that immersive VR may have educational value for preparing future nurses to respond to high‐pressure clinical and disaster environments.

### 5.1 Immersive VR as a Mechanism for Transforming Abstract Risk Into Embodied Understanding

A key contribution of this study is the identification of immersive VR as a mechanism that transforms abstract disaster knowledge into embodied and emotionally salient understanding. Participants reported intense emotional responses, including fear, helplessness and urgency, which heightened their perception of vulnerability and made disaster risks more personally relevant. These findings are consistent with previous studies indicating that experiential learning enhances situational awareness and risk perception by increasing emotional engagement [14, 15]. From a theoretical perspective, this process aligns with Protection Motivation Theory (PMT), in which perceived severity and vulnerability (threat appraisal) play central roles in motivating protective behaviours [16]. The present findings further suggest that immersive VR may facilitate psychological preparedness by allowing learners to experience emotionally challenging situations within psychologically safe educational environments. In critical care and emergency nursing education, simulation‐based learning is increasingly recognised as an important educational strategy for developing clinical judgement and decision‐making skills in complex and rapidly evolving clinical situations [21, 22]. Immersive VR may therefore provide opportunities for nursing students to develop emotional and cognitive readiness for disaster‐related critical care situations. Furthermore, the findings support the notion that emotional and sensory engagement are critical drivers of risk perception, particularly for individuals without prior disaster experience, as risk perception is strongly influenced by affective and experiential factors [23]. By simulating realistic disaster scenarios, VR enables individuals to internalise risks as personally relevant, thereby addressing limitations associated with traditional didactic disaster education.

### 5.2 Explaining the Awareness–Action Gap: The Role of Coping Appraisal and Self‐Efficacy

Despite enhanced risk perception, participants reported difficulty translating awareness into concrete preparedness actions. This finding reinforces the widely recognised ‘risk perception paradox’, whereby increased awareness does not necessarily lead to behavioural change [9]. Within the PMT framework, this gap may be explained by insufficient coping appraisal, particularly low self‐efficacy and uncertainty regarding appropriate actions. While VR effectively strengthened threat appraisal (perceived severity and vulnerability), participants expressed doubts about their ability to respond effectively in real‐world disaster situations. This is consistent with previous research demonstrating that preparedness behaviour is influenced not only by risk perception but also by perceived capacity to act ([20]; Grothmann & Reusswig, 2006). Importantly, the findings indicate that immersive emotional experiences alone may not automatically strengthen preparedness behaviours or response confidence. Although emotional engagement may enhance awareness, excessive psychological arousal could potentially contribute to anxiety, helplessness or cognitive overload in some learners. This suggests that immersive VR should be carefully integrated with reflective learning opportunities, debriefing and skills‐based preparedness training to support adaptive coping and behavioural translation.

In addition, participants described habituation to repeated disaster information, leading to diminished urgency. This aligns with findings that risk perception is dynamic and shaped by familiarity, experience and contextual exposure [9]. These results suggest that interventions focusing solely on increasing awareness may have limited impact unless accompanied by strategies that enhance self‐efficacy and actionable knowledge. The imbalance observed between threat appraisal and coping appraisal may be particularly relevant in disaster and critical care education, where healthcare professionals are often expected to function effectively under uncertainty and rapidly changing conditions. Educational strategies that combine immersive simulation with practical preparedness training may therefore be important for strengthening both emotional readiness and perceived response capability.

### 5.3 From Individual Awareness to Collective Preparedness: Implications for Disaster Risk Reduction

An important extension of previous research is the finding that VR experiences fostered not only individual risk awareness but also recognition of the vulnerability of others and the need for support systems. Participants reflected on the challenges faced by older adults and other vulnerable populations, highlighting the social dimension of disaster preparedness. This finding aligns with DRR frameworks emphasising that preparedness is not solely an individual responsibility but a collective process involving communities, institutions and healthcare systems [1]. Risk perception is shaped by both individual and social factors, and effective DRR requires coordinated action across multiple levels [10]. Participants also recognised the potential responsibilities of healthcare professionals in supporting vulnerable populations during disasters. Such reflections may be important for developing professional identity and ethical awareness among future nurses who may later participate in emergency and critical care responses during disasters. By enabling users to experience diverse perspectives and situational constraints, VR may facilitate empathy, social awareness and collective responsibility. These findings suggest that immersive technologies can contribute to strengthening not only individual preparedness but also community‐level resilience, which is a central goal of DRR.

### 5.4 Implications for Disaster Preparedness and Critical Care Education

This study provides several important implications for disaster preparedness and critical care nursing education. First, immersive VR can serve as an effective educational tool for strengthening emotional engagement, situational awareness and disaster risk perception among future healthcare professionals. Second, immersive simulation‐based learning may support psychological preparedness and decision‐making in uncertain and high‐pressure situations relevant to disaster and critical care contexts. Third, the findings suggest that VR‐based disaster education should not function as a stand‐alone intervention. Rather, immersive learning may be most effective when combined with reflective debriefing, collaborative simulation activities and practical preparedness training that strengthen coping appraisal and self‐efficacy. Such multimodal approaches may help bridge the awareness–action gap identified in the present study. Integrating VR with reflective learning, simulation‐based training and collaborative preparedness activities may facilitate the translation of awareness into sustained action. These findings are consistent with emerging evidence that multimodal and participatory approaches are more effective in promoting preparedness behaviours [13]. Finally, as climate change continues to increase the frequency and complexity of disasters globally, healthcare systems will require healthcare professionals who are psychologically and operationally prepared for disaster response. Immersive VR‐based education may therefore have broader relevance for strengthening healthcare system resilience and future disaster response capacity within critical care and emergency nursing contexts.

**FIGURE 1:**
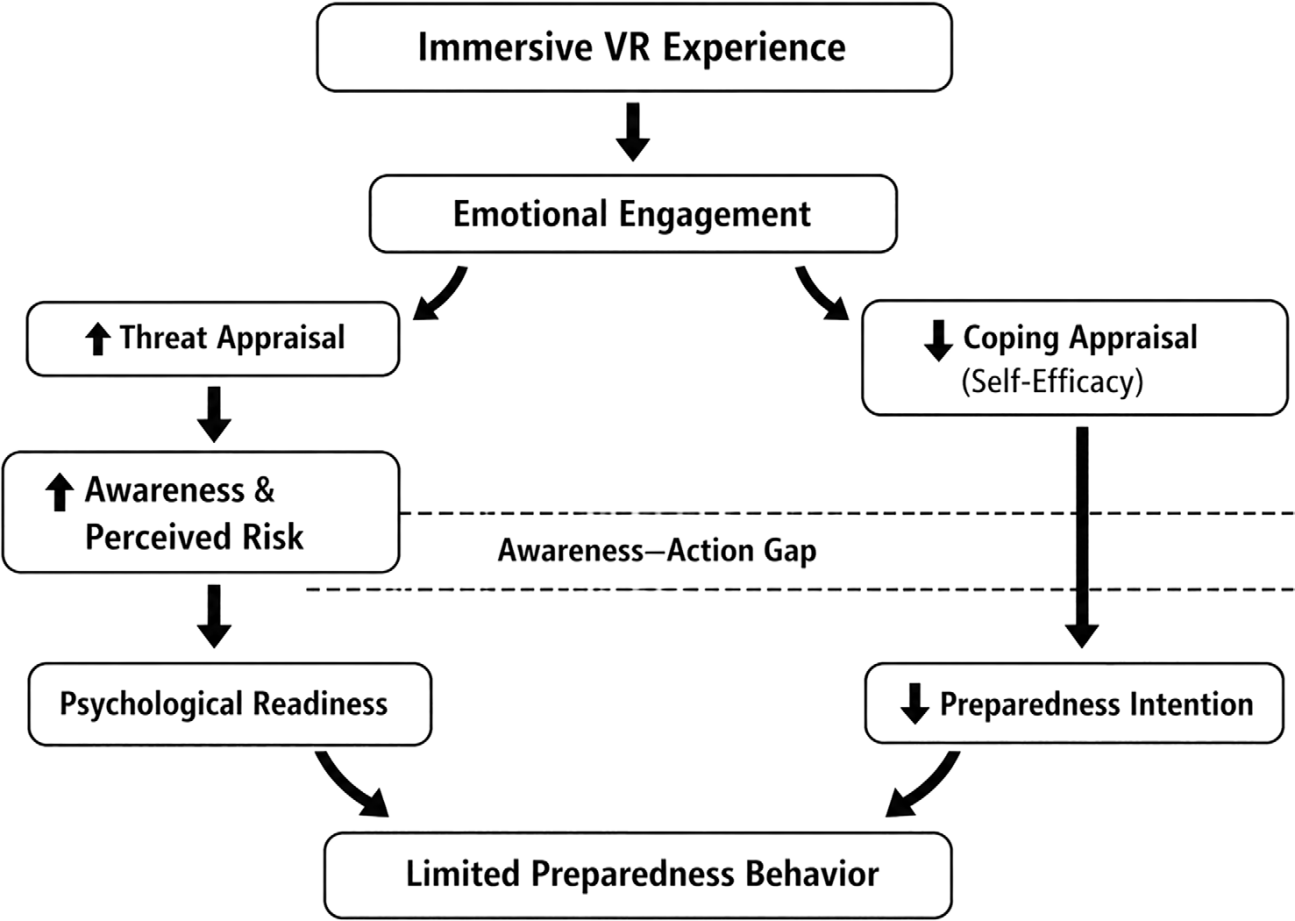
Mechanisms through which immersive VR influences disaster risk perception and preparedness: The awareness–action gap.

A conceptual model illustrating the mechanisms through which immersive VR influences disaster risk perception and preparedness, including the awareness–action gap identified in this study, is presented in Figure 1.

## 6 Limitations

Several limitations should be acknowledged. First, the study was conducted at a single university in Japan and included nursing students who voluntarily participated, which may limit the transferability of the findings to other student populations, educational and cultural contexts, or disaster‐prone regions. Future studies should include more diverse participant groups, including non‐healthcare populations, to enhance generalisability within disaster preparedness and DRR contexts.

Second, the study focused on short‐term experiential responses immediately following the VR simulation. As such, the findings do not capture whether enhanced risk perception and preparedness intentions translate into sustained behavioural change over time. Longitudinal studies are needed to evaluate the lasting impact of immersive interventions on actual preparedness behaviours.

Third, the VR scenarios were limited to specific hazard types (e.g., floods and landslides) and may not reflect the complexity and variability of real‐world disaster contexts. Different hazard types, levels of realism and interactive elements may influence risk perception and behavioural responses differently.

In addition, although immersive VR promoted emotional engagement, the simulated experiences may not fully reproduce the interpersonal complexity, ethical uncertainty and clinical demands encountered in actual disaster and critical care situations. Therefore, caution is needed when translating simulated preparedness into assumptions regarding real‐world clinical performance.

Finally, as with all qualitative research, interpretive bias cannot be completely eliminated. Although efforts were made to enhance rigour through researcher triangulation and systematic analysis, the findings should be interpreted within the context of the study design and analytical approach.

## 7 Future Directions

Future research should extend beyond short‐term experiential outcomes to examine whether VR‐based disaster simulations lead to sustained behavioural change and actual preparedness actions over time. Longitudinal and intervention‐based studies are needed to evaluate how immersive technologies can effectively bridge the awareness–action gap within real‐world disaster preparedness and DRR contexts.

Future studies should also examine how immersive VR can be integrated into emergency and critical care nursing education programmes, including disaster triage training, emergency response simulations and interprofessional preparedness exercises. Exploring how immersive learning influences psychological readiness, clinical judgement and response confidence in high‐pressure healthcare situations may further clarify its educational value.

In addition, future studies should explore the integration of VR with complementary strategies that enhance coping appraisal, such as skills‐based training, reflective learning and collaborative simulations. Investigating how different combinations of interventions influence self‐efficacy and behavioural translation will be critical for optimising preparedness programmes.

As climate‐related disasters continue to affect healthcare systems globally, further research examining scalable and sustainable immersive educational approaches may contribute to strengthening healthcare workforce preparedness and healthcare system resilience.

## 8 Conclusion

Immersive VR‐based disaster simulations may strengthen disaster risk perception and preparedness awareness among nursing students by transforming abstract disaster risks into emotionally salient and personally relevant experiences. However, heightened awareness alone may be insufficient to translate risk recognition into preparedness action. Integrating immersive VR with reflective learning, skills‐based training and collaborative simulation may strengthen coping appraisal, self‐efficacy and preparedness competencies relevant to disaster and critical care nursing. As climate‐related disasters continue to affect healthcare systems globally, further research examining scalable and sustainable immersive educational approaches may contribute to strengthening healthcare workforce preparedness and healthcare system resilience.

## Author Contributions

**K.M.:** conceptualization, methodology, supervision, and writing – review and editing. **M.Y.:** data curation, investigation and writing – original draft. **Y.H.:** resources and technical support (VR content provision and VR viewing support). All authors have read and approved the final manuscript. All authors agree to be accountable for all aspects of the work.

## Funding

The authors have nothing to report.

## Ethics Statement

Ethical approval to conduct the study was obtained through a simplified ethical review process at Okayama University before data collection. As required by institutional procedures for external dissemination of research findings, the study and its findings subsequently underwent formal review, and ethical approval for external dissemination was obtained post‐hoc from the Ethics Committee of Okayama University Graduate School of Health Sciences (Approval No. OUHS2025‐0087F, 26 March 2026).

## Consent

Informed consent was obtained from all individual participants included in the study.

## Conflicts of Interest

Yoshiyuki Haruna is the president of Snowlion Inc., the company that developed the immersive VR disaster content used in this study. He contributed to the development and technical implementation of the VR content and was present during the VR sessions solely to provide technical support for the operation of the VR equipment. He was not involved in conducting the semi‐structured interviews, qualitative data analysis, interpretation of the findings, or development of the conclusions. The other authors declare no conflicts of interest.

## Supporting information


**Data S1:** Supporting Information.

## Data Availability

The data that support the findings of this study are not publicly available due to privacy and ethical restrictions. De‐identified data may be available from the corresponding author upon reasonable request and with approval from the institutional ethics committee.
