# Supplementary material for: Bridging the Awareness–Action Gap in Disaster Preparedness: A Qualitative Study of Immersive Virtual Reality Learning Among Nursing Students
Source: Nurs Crit Care. 2026 Sep 24;31(6):e70681. doi: 10.1111/nicc.70681 (PMC13612933; doi:10.1111/nicc.70681)
Supplement: Supplementary file 1 [file NICC-31-0-s001.docx]

Supplementary Material

Interview Guide

The following questions were used as a semi-structured interview guide to explore participants’ experiences, perceptions, and behavioral intentions related to disaster risk and preparedness following the VR simulation.

Participant characteristics

-Age:

-Gender:

-Place of residence:

-Prior disaster experience (Yes/No; if yes, please describe):

Interview Questions

1. Can you describe what you usually think about disasters in your daily life?
2. Do you usually take any actions or preparations related to disaster preparedness?
3. What aspects of the VR disaster experience left a strong impression on you?
4. What kind of emotions did you experience during the VR simulation?
5. How did you feel when imagining yourself in a real disaster situation?
6. Did your understanding or perception of disasters change after the VR experience? If so, how?
7. Before the VR experience, how would you describe your awareness and preparedness regarding disasters?
8. How did the VR experience influence your awareness and preparedness, if at all?
9. In what ways, if any, did the VR experience influence your motivation or intention to take preparedness actions?
10. What actions do you think you should take to protect yourself and your family in a disaster?
11. Do you think experiencing disaster situations through VR is useful? Please explain why.
12. What improvements would you suggest to make the VR disaster experience more effective?
13. What kind of education or training would help you think more deeply about disaster preparedness?

Additional probing questions were used as needed to elicit deeper reflections and clarify participants’ responses.
